# Supplementary material for: Amino acid 17 in QRDR of Gyrase A plays a key role in fluoroquinolones susceptibility in mycobacteria
Source: Microbiol Spectr. 2023 Oct 13;11(6):e02809-23. doi: 10.1128/spectrum.02809-23 (PMC10715211; doi:10.1128/spectrum.02809-23)
Supplement: Table S1 — Oligonucleotides used in this study. [file spectrum.02809-23-s0001.docx]

Supplementary Materials

**Amino acid 17 in QRDR of Gyrase A plays a key role in fluoroquinolones susceptibility in Mycobacteria**

Shuai Wang ^a,b,c,d†^, Jingran Zhang ^a,c,d,e†^, H.M. Adnan Hameed ^a,b,c,d^, Jie Ding ^a,c,d,f^, Ping Guan^g^, Xiange Fang ^a,b,c,d^, Jiacong Peng^c,h^, Biyi Su^g^, Shangming Ma^g^, Yaoju Tan^g^, Gregory M. Cook^i,j^, Guoliang Zhang^k^, Yongping Lin^c,h^, Nanshan Zhong^c,h,l^, Jinxing Hu^g*^, Jianxiong Liu^g*^, Tianyu Zhang ^a,b,c,d*^

^a^ State Key Laboratory of Respiratory Disease, Guangzhou Institutes of Biomedicine and Health, Chinese Academy of Sciences, Guangzhou 510530, China

^b^ University of Chinese Academy of Sciences, Beijing 100049, China

^c^ Guangdong-Hong Kong-Macao Joint Laboratory of Respiratory Infectious Diseases, Guangzhou Institutes of Biomedicine and Health, Chinese Academy of Sciences, Guangzhou 510530, China

^d^ China-New Zealand Joint Laboratory on Biomedicine and Health, Guangzhou, 510530, China

^e^ School of Life Sciences, University of Science and Technology of China, Hefei, Anhui 230027, China

^f^ Institutes of Physical Science and Information Technology, Anhui University, Hefei, Anhui 230601, China

^g^ State Key Laboratory of Respiratory Disease, Guangzhou Chest Hospital, Guangzhou, China

^h^ State Key Laboratory of Respiratory Disease, National Clinical Research Center for Respiratory Disease, The National Center for Respiratory Medicine, The First Affiliated Hospital of Guangzhou Medical University, Guangzhou, 510230, China

^i^ Department of Microbiology and Immunology, School of Biomedical Sciences, University of Otago, Dunedin, New Zealand

^j^ Maurice Wilkins Centre for Molecular Biodiscovery, The University of Auckland, Private Bag, Auckland, New Zealand

^k^ National Clinical Research Center for Infectious Diseases, Guangdong Provincial Clinical Research Center for Tuberculosis, Shenzhen Third People's Hospital，Shenzhen, 518055, China

^l^ Guangzhou Laboratory, Bio-Island, Guangzhou 510320, China

^*^Correspondence: Tianyu Zhang, [zhang_tianyu@gibh.ac.cn](mailto:zhang_tianyu@gibh.ac.cn); Jianxiong Liu, ljxer64@qq.com; Jinxing Hu, [hujinxing2000@163.com](mailto:hujinxing2000@163.com;).

^†^ These authors contributed equally to this work.

**Table S1.** Oligonucleotides used in this study.

| Name | Sequence (5’-3’) | Description |
| --- | --- | --- |
| T82-crRNA-F | ATCCGAGACCATGGGTAACTATCAA | crRNA for T7A/A17G+T7A/S17A editing |
| T82-crRNA-R | AGCTTTGATAGTTACCCATGGTCTCGGATT |  |
| A92-crRNA-F | ATCGACACCCTCGTGCGTATGGA | crRNA for A17G/A17S editing |
| A92-crRNA-R | AGCTTCCATACGCACGAGGGTGTCGATCT |  |
| A92S-R | GGGCCATACGCACGAGGGTGTCGTATATGGAGGAGTCACCGTGGGGGTGATAGTTACCC | recombinogenic oligonucleotides for A17S editing |
| A92G-R | GGGCCATACGCACGAGGGTGTCGTATATGGATCCGTCACCGTGGGGGTGATAGTTACCC | recombinogenic oligonucleotides for A17G editing |
| T82A-R | CGTCACCGTGGGGGTGATAGTTACCCATTGCCTCGGCAACCGAGCGCGCCGACTTGGCA | recombinogenic oligonucleotides for T7A editing |
| A92G+T82A-UF | ctcgaggtcgacggtatcgatGATACCGCGACGGCCGCC | construct a plasmid containing A17G + T17A mutation homologous arms |
| A92G+T82A -UR | ATGGATCCGTCACCGTGGGGGTGATAGTTACCCATTGCCTCGGCAACCGAGCGC |  |
| A92G+T82A -DF | CGAGGCAATGGGTAACTATCACCCCCACGGTGACGGATCCATCTACGACACCCTCGT |  |
| A92G+T82A -DR | cgctctagaactagtggatccCTGCTCGGCGATGGAGGT |  |
| S92A-UR | ATAGTTACCCATGGTCTCGGCTACCGAGCGCGC | construct a plasmid containing S17A mutation homologous arms |
| S92A-DF | CCGAGACCATGGGTAACTATCACCCCC |  |
| gyrA-UD-F | GATACCGCGACGGCCGCC | amplify homologous arm fragments |
| gyrA-UD-R | CTGCTCGGCGATGGAGGT |  |
